# High resolution microscopy reveals the nuclear shape of budding yeast during cell cycle and in various biological states

**DOI:** 10.1242/jcs.188250

**Published:** 2016-12-15

**Authors:** Renjie Wang, Alain Kamgoue, Christophe Normand, Isabelle Léger-Silvestre, Thomas Mangeat, Olivier Gadal

**Affiliations:** 1 Laboratoire de Biologie Moléculaire Eucaryote, Centre de Biologie Intégrative (CBI), Université de Toulouse, CNRS, UPS, Toulouse 31000, France; 2 Laboratoire de Biologie Cellulaire et Moléculaire du Contrôle de la Prolifération, Centre de Biologie Intégrative (CBI), Université de Toulouse, CNRS, UPS, Toulouse 31000, France

**Keywords:** Localization microscopy, Nuclear pore complex, Nuclear geometry, Super resolution microscopy

## Abstract

How spatial organization of the genome depends on nuclear shape is unknown, mostly because accurate nuclear size and shape measurement is technically challenging. In large cell populations of the yeast *Saccharomyces cerevisiae*, we assessed the geometry (size and shape) of nuclei in three dimensions with a resolution of 30 nm. We improved an automated fluorescence localization method by implementing a post-acquisition correction of the spherical microscopic aberration along the *z*-axis, to detect the three dimensional (3D) positions of nuclear pore complexes (NPCs) in the nuclear envelope. Here, we used a method called NucQuant to accurately estimate the geometry of nuclei in 3D throughout the cell cycle. To increase the robustness of the statistics, we aggregated thousands of detected NPCs from a cell population in a single representation using the nucleolus or the spindle pole body (SPB) as references to align nuclei along the same axis. We could detect asymmetric changes of the nucleus associated with modification of nucleolar size. Stereotypical modification of the nucleus toward the nucleolus further confirmed the asymmetric properties of the nuclear envelope.

## Abstract (precis)

**Summary:** This novel method to explore 3D geometry of the nuclear envelope with enhanced resolution and post-acquisition correction of *z*-axis aberration revealed increased NPC density near the SPB and the nucleolus.

## INTRODUCTION

Structural organization of the genome is a key determinant in all genome transactions, including transcription and genome stability. In eukaryotic cells in interphase, genomic DNA is segregated away from the cytoplasm by the nuclear envelope. Components of the inner nuclear membrane (INM) or nuclear pore complexes (NPCs) are crucial players in the spatial regulation of gene expression or genome stability (Towbin et al., 2009). Modification of the nuclear radius or altered sphericity affects the ability of loci to interact with NPCs and INM (Zhao et al., 2016). The confinement of the genome of *S. cerevisiae* in the nucleus persists throughout the cell cycle as they have a closed mitosis. Few structural features are involved in the inner organization of the budding yeast nucleus in interphase: the spindle pole body (SPB), centromeres, telomeres and the nucleolus. The SPB, the budding yeast microtubule-organizing center, is embedded into the nuclear envelope. With the exception of a short time window after centromere replication, the SPB anchors each centromere by means of a microtubule spindle during the entire cell cycle (Winey and O'Toole, 2001), whereas telomeres are associated with the nuclear envelope (Taddei et al., 2010). In exponentially growing cells, nuclear volume is subdivided into two thirds containing the nucleoplasm and one third containing the nucleolus (Léger-Silvestre et al., 1999). With the SPB and the nucleolus being diametrically opposed in interphase (Yang et al., 1989), the SPB, the nuclear center and the nucleolar centroid define a central axis around which chromosomes are organized. This axis enabled the design of chromatin models as space-filling polymer, which accurately recapitulate most of the known features of the genome organization (Tjong et al., 2012; Wong et al., 2012). Importantly, Alber's laboratory has shown that an accurate simulation of chromosome positioning largely depends on constrains imposed by the shape of the nucleoplasm (Tjong et al., 2012). Therefore, nuclear volume and shape need to be precisely defined to accurately explore eukaryotic genome organization.

The nucleus in budding yeast is often described as a sphere of radius ∼1 µm, which ignores described variations of size: the median nuclear volume can vary up to twofold between yeast strains (Berger et al., 2008); carbon source has major impact on the nuclear size (Jorgensen et al., 2007); and each yeast nucleus undergoes a twofold increase in volume from G1 to S phase (Jorgensen et al., 2007; Winey et al., 1997). Additionally, the budding yeast nucleus is not a perfect sphere and size determination cannot always rely on spherical approximation (Zhao et al., 2016). The vacuole is also known to modify nuclear shape (Severs et al., 1976). During closed mitosis, the nucleus adopts a number of non-spherical conformations; the microtubule spindle cycle modifies nuclear shape (Yeh et al., 1995) and bud constriction constrains nuclear morphology (Boettcher et al., 2012). The nuclear division process is asymmetric; the mother cell nucleus is one half larger than the daughter cell nucleus (Heun et al., 2001). In the nucleus, the SPB and nucleolus are known to impact on nuclear shape. During mitosis, the SPB can affect locally nuclear envelope sphericity (Koning et al., 1993). In interphasic cells growing exponentially in medium containing glucose, the nucleolus is described as a crescent-shaped nuclear domain flanking the nuclear envelope. During cell cycle delay (S or G2), nuclear envelope expansion is constrained toward the nucleolus (Witkin et al., 2012). All these observations highlight the highly dynamic nuclear envelope and the variability of nuclear size and shape (Stone et al., 2000; Webster et al., 2009). Accurate determination of the nuclear envelope position using fluorescence microscopy is technically challenging and is mostly performed in two dimensions (2D) (Dultz et al., 2016). Recent techniques have been proposed to explore the nuclear geometry in 3D (Zhao et al., 2016).

Here, we developed ‘NucQuant’, an optimized automated image analysis algorithm, accurately interpolating the nuclear envelope position in a large number of cell nuclei in 3D. Super-resolution fluorescence localization microscopy (e.g. PALM, FPALM, STORM) is now a well-established concept used to break resolution barriers in fluorescence microscopy: 200 nm in *x*–*y* and ∼500 nm in the *z*-axis (Nelson and Hess, 2014). Localization microscopy (e.g. PALM, FPALM, STORM) measures the position of isolated objects, single molecules or point-like structures, with an uncertainty of a few tens of nanometers. Isolated point-like structures can be fitted with the characteristic Gaussian distribution of fluorescence around local maxima approximating the point spread function of the optical setup (Thomann et al., 2002). GFP-tagged NPC components appear as a typical punctate ring staining the nuclear envelope (Wimmer et al., 1992). This bright staining originates from a high number of nucleoporins per NPC (up to 64) and the presence of 60 to 180 NPCs per nucleus (Rout et al., 2000; Winey et al., 1997). The punctate staining is caused by the non-random distribution of NPC within the nuclear envelope (Winey et al., 1997), resulting in local clusters of NPCs. Therefore, adjacent NPCs, convolved by optical set-up, appear as punctuated bright spots within the nuclear envelope. Therefore, GFP-tagged nucleoporins represent ideal point-like structures to follow the nuclear envelope shape and size by localization microscopy (Berger et al., 2008). We localized fluorescently labeled NPC and corrected detection bias resulting from optical spherical aberration along the *z*-axis to accurately compute an approximation of the nuclear envelope in 3D. This approach allowed us to precisely measure the size and shape of the yeast nucleus throughout the cell cycle or in cells growing on different carbon sources; we could recapitulate the considerable level of plasticity of the nuclear envelope. Using the nucleolus or SPB as a spatial landmark to align nuclei along the same axis and aggregate thousands of detected NPCs from a cell population in a single representation, we could evaluate the NPC density along the nuclear envelope in different physiological conditions. We detected a low NPCs density in the nuclear envelope at the nucleolar–nucleoplasmic interface and stereotypical modifications of the nuclear envelope correlated with nucleolar size variations.

## RESULTS

### Localization microscopy using confocal fluorescence imaging of nuclear pore complexes is a super-resolution microscopic technique

‘Localization microscopy’ relies on detection of the centroid of point-like structures, and is classically not limited by diffraction, but by the signal-to-noise ratio of individual detections. To label NPC, we fused GFP or mRFP dimers with abundant NPC components (Nup49, Nup57, Nup2 or Nup159). We developed an algorithm called ‘NucQuant’, adapted from a previously described automated image analysis pipeline using spinning-disk fluorescence microscopy and MATLAB codes (Berger et al., 2008). We could detect a median of 22 peripheral spots per cell nucleus. Each detected spot should roughly correspond to a group of three to nine closely spaced NPCs, hereafter called cNPCs (Winey et al., 1997). Importantly, detection of 22 spots per nucleus provided sufficient connecting points to evaluate the nuclear envelope position in 3D.

**Fig. 1.**
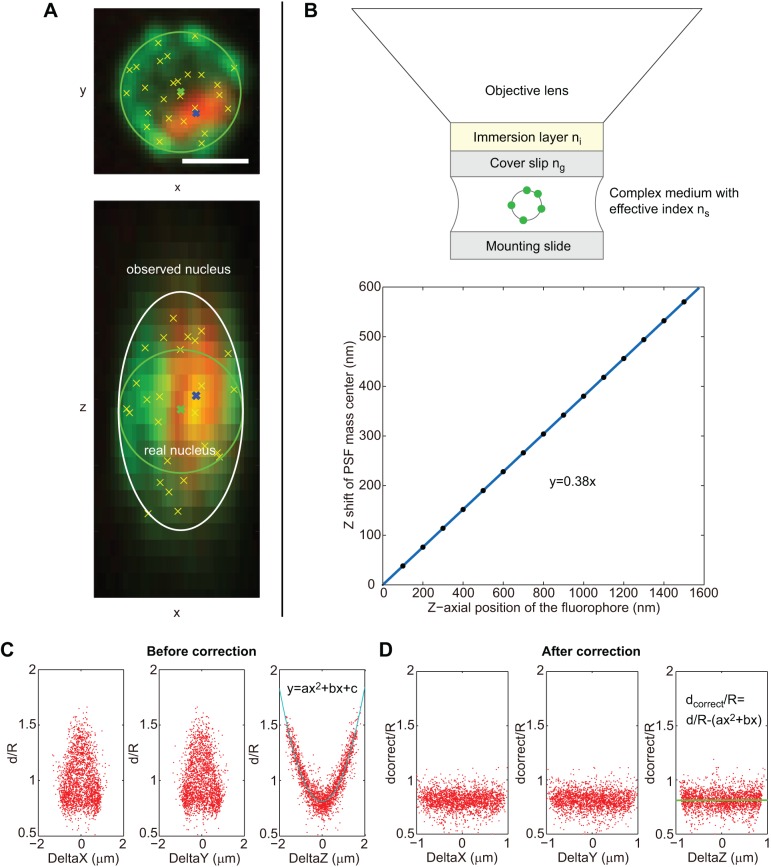
**Detection and correction of the aberrations along the *z* axis.** (A) Yeast nucleus in exponential phase with nuclear pores labeled in green and the nucleolus in red (maximum intensity projections of a 3D image stack in *x*–*y* plane and *x*–*z* plane). Yellow crosses show detected NPCs, green crosses show the nucleus center, blue crosses show nucleolus centroid. Green circles show the expected edge of the nucleus and white ellipse shows the detected edge. Strain yCNOD99-1a. Scale bar: 1 μm. (B) Immersion layer refractive index=1.51, cover slip 170 μm and refractive index=1.51, sample refractive index=1.38. Objective lens: NA=1.4×100, lambda=520 nm. Linear *z*-level shift of PSF mass center and the real *z*-axial position of the fluorophore. (C) The normalized distance distribution of the detected NPCs to the nuclear center along *x*-level, *y*-level and *z*-level before correction of the aberration along *z* axis. d, distance of NPCs to the nuclear center; R, radius of each nucleus. Strain yCNOD99-1a, a=0.26, b=0.0029, c=0.81. (D) The normalized distance distribution along *x*-level, *y*-level and *z*-level after correction of the aberration along *z* axis. d_correct_=corrected distances of NPCs to the nuclear center.

Refractive index mismatch between optical setup and biological object is known to generate spherical aberration along the *z*-axis (Fig. 1A,B). We measured and corrected this detection bias in our measurements. For exponentially growing cells in glucose, electron microscopy and X-ray tomography established that the yeast nucleus in interphase is mostly spherical (Larabell and Le Gros, 2004; Murata et al., 2014; Wei et al., 2012; Winey et al., 1997). We analyzed ∼1000 round cell nuclei and could generate a statistically robust dataset of ∼20,000 cNPC detections (Fig. 1C). The nuclear sphericity allowed measurement of detection bias in our localization dataset. In a sphere, distances between detected cNPCs and the nuclear center are the same in each dimension (*x*, *y* and *z*) (Fig. S1A,C). Simulating the elongation along the *z*-axis (Fig. S1A,B) modified the distribution of normalized distances between detected cNPCs and nuclear center along the *x*, *y* and *z* axes (compare Fig. S1C and D). Similar over-estimation along the *z*-axis was clearly detected in our experimental measurement (Fig. 1C). In our experimental dataset, post-acquisition correction of localized cNPCs was performed as suggested previously (see Materials and Methods; Fig. S1E) (Cabal et al., 2006). This correction was calculated using round nuclei for each cell population analyzed, and was subsequently applied to the entire population (including non-round nuclei; Fig. S1F). Note that spherical aberration was always detectable, but was specific for different objectives or microscope setups (confocal laser scanning – versus laser spinning disk – microscopes) (Fig. S1G,H).

**Fig. 2.**
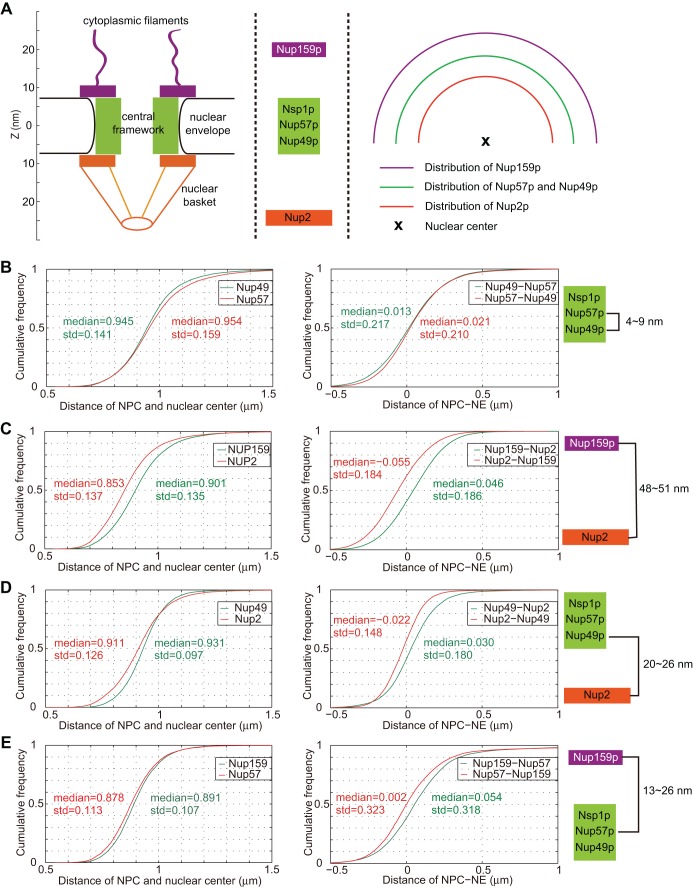
**Resolution of NucQuant after correction of the aberration along the *z* axis.** (A) Nuclear pore complex (NPC) architecture and nucleoporin localization in the NPC. (B–E) Cumulative frequency of distances to the nuclear center (left panels) and of distances to the fitted ellipsoid approximation of the nuclear envelope (right panels) using GFP or mRFP-tagged nucleoporins. (B) GFP–Nup49 and mRFP–Nup57, strain yRW3-1a. (C) GFP–Nup159 and mRFP–Nup2, strain yRW7-1a. (D) GFP–Nup49 and mRFP–Nup2, strain yRW4-1a. (E) GFP–Nup159 and mRFP–Nup57, strain yRW8-1a.

To evaluate the resolution of our detection method after spherical aberration correction, we made use of the known distribution of nucleoporins in NPC near the central channel (Nsp1 complex subunits: Nup49 and Nup57), toward the nuclear basket (Nup2) and localized in the cytoplasmic filaments (Nup159) (Fig. 2A) (Alber et al., 2007; Dilworth et al., 2001; Grandi et al., 1995; Rout et al., 2000). To analyze the distribution of detected cNPCs, we computed their radial distances to the nuclear center. Simultaneously labeling two nucleoporins respectively with GFP or mRFP, we detected ∼20,000 cNPCs in each color from the population of cell nuclei. Radial distribution of detected cNPCs was plotted as cumulative frequency of either distances to the nuclear center or a fitted ellipsoid approximation of the nuclear envelope using GFP or mRFP signals (Fig. 2B–E). As expected, median distances (to the nuclear center or to the fitted nuclear envelope) between constituents of the Nsp1 subcomplex (Nup49–GFP and Nup57–mRFP) were equivalent (+4 to +9 nm; Fig. 2B). Median distance separating nuclear basket (Nup2–mRFP; intranuclear structure) to cytoplasmic filaments (Nup159–GFP; most distant structure to nuclear center) was from +48 to +51 nm (Fig. 2C). Adding the distance measured from nuclear basket to central channel (+20 to +26 nm) to the distance from central channel to cytoplasmic filaments (+13 to +26 nm) allowed *in vivo* determination of the cNPC length perpendicular to the nuclear envelope (from +33 to 52 nm) (Fig. 2D,E). This distance, measured *in vivo*, is largely comparable with the 50 nm separating the nuclear basket from the cytoplasmic filaments as measured by transmitted electron microscopy (TEM) after immuno-detection of Protein-A-tagged nucleoporins from purified nuclear envelope (Alber et al., 2007). Furthermore, *in vivo* fluorescently tagged Nup159 and the nucleoporin Nup60, which colocalized with Nup2 on the inner side of NPC, were shown to be separated by 60 nm using structured illumination microscopy (Guet et al., 2015).

We concluded that localization of cNPCs in the nuclear envelope can be used to achieve robust super-resolution detection with sufficient accuracy to distinguish longitudinal position of nucleoporins within NPC. Therefore, we used the accuracy of cNPC detections to explore the nuclear envelop in 3D.

### 3D nuclear envelope model for complex-shaped nuclei along the cell cycle

**Fig. 3.**
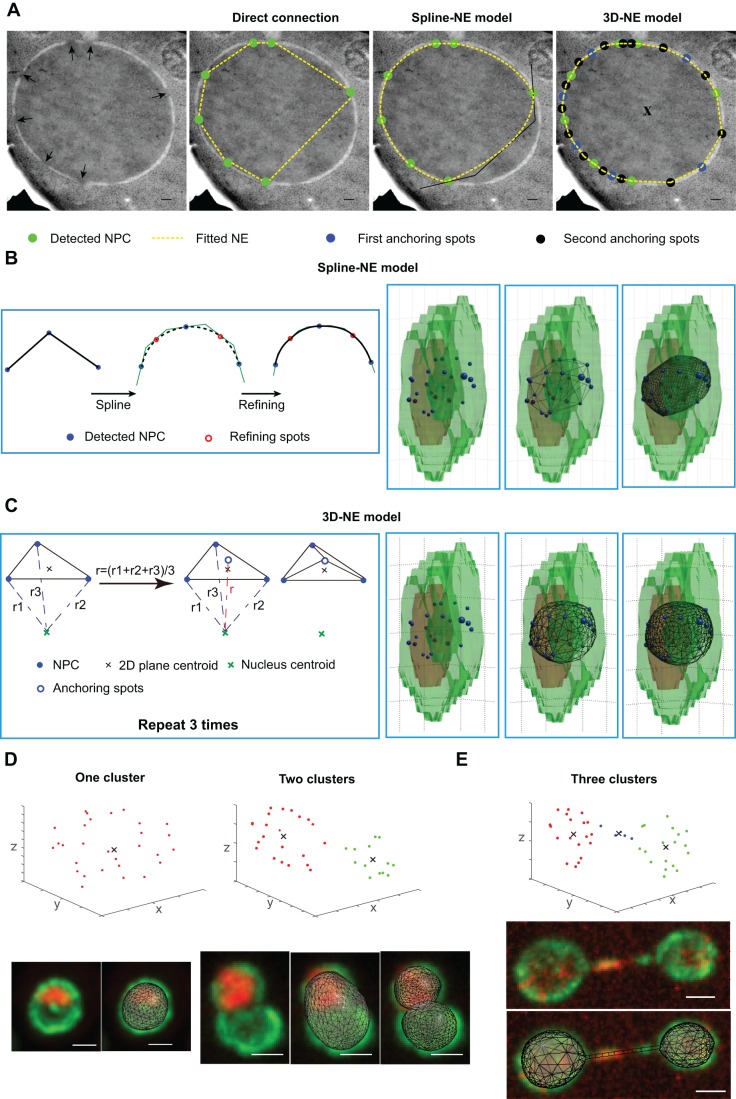
**Extrapolation of nuclear envelope using detected NPCs.** (A) 2D models building nuclear envelopes are represented onto electron microscopic micrographs of nuclear sections on which the positions of NPCs are visually detected (black arrows in the left panel). Strain BY4741. In the second image, the nuclear envelope is built by connecting adjacent NPCs. In the third image, the nuclear envelope is built by spline interpolation. In the right panel, the nuclear envelope is fitted by generating anchoring spots in nuclear envelope. Scale bars: 100 nm. (B,C) Based on the 3D confocal microscopic images, we could detect the NPC positions (blue spheres). Strain yCNOD99-1a. Using the spline-NE model (B), we refined the connection to get a smooth nuclear envelope. Red circles represent the spots that were used to refine the connection. 3D-NE model (C) generates additional anchoring spots (blue empty circle) to get an accurate extrapolation of the nuclear envelope. (D) The fitted nuclear envelope based on 3D-NE model for the nuclei characterized by one (left) or two (right) NPCs clusters. Upper graphs: *x*, *y*, *z* coordinates of detected NPCs; black cross, centroid of detected cluster(s); cluster 1 in red, cluster 2 in green. Strain yCNOD99-1a. (E) The fitted nuclear envelope based on 3D-NE model for the anaphase nuclei characterized using three NPCs clusters. Cluster 3 in blue. Strain yCNOD203-1a. Scale bars in D,E: 1 µm.

An asynchronous cell population contains cells in all cell cycle phases. The most abundant cells are in G1–S with quasi-round nuclei that were analyzed in previous works thanks to a spherical or ellipsoid fitting (Berger et al., 2008; Fig. 2). To improve nuclear envelope fitting, the nuclear size and shape of quasi-round nuclei were explored using the position of cNPCs. The high number of detected cNPCs per nucleus allowed a precise interpolation of the nuclear envelope geometry. We generated a 3D model of the nuclear envelope using detected cNPCs. We also tested model prediction against simulated NPC positions for a sphere or an ellipsoid (Fig. S2). Nuclear envelope shape in 2D was detected at very high resolution in electron microscopic micrographs of sections of cells fixed by high-pressure freezing followed by freeze substitution. This cell preparation resulted in optimal morphological preservation of the nuclear envelope (Fig. 3A). Direct connection of adjacent detected cNPCs in 2D resulted in a non-accurate estimation of the nuclear envelope (Fig. 3A). Then, we computed a smooth estimation of nuclear envelope by connecting adjacent detected cNPCs using spline interpolation (spline-NE model) (see Materials and Methods; Fig. 3A,B). Using this approach, nuclear surfaces and volumes were systematically underestimated (Fig. 3A,B; Fig. S2A). In this spline-NE model, detected cNPCs must be the most distant points of the nuclear envelope to the nuclear center. This geometric constraint could trigger bias in the nuclear envelope approximation on yeast sections. To prevent such bias, we generated additional anchoring points of the nuclear envelope using the three closest cNPCs (Fig. 3A,C; see Material and Methods). Using simulated NPC positions, this method, called the 3D-NE model, slightly overestimated nuclear size for small numbers of NPCs (<20), but was more accurate than spline-NE for high numbers of NPCs (Fig. S2B). From the 3D-NE model, we could extract the surface (S_n_) and volume (V_n_) of each nucleus accurately in interphasic cells with quasi-round nuclei (Fig. 3C, right panel).

Cells in G2 or M displayed non-spherical nuclei with complex shapes ranging from elongated to constricted, sausage- or hourglass-shaped structures (Yeh et al., 1995). In non-spherical nuclei, the spatial coordinates of cNPCs distributed as more than one cluster (Fig. 3D, upper graphs). The 3D-NE model using only one cluster was indeed not accurate and resulted in cNPC at the constriction ring appearing inside the fitted nucleus (Fig. 3D, lower mid-right panel). On splitting cNPCs into two clusters, the 3D-NE model performed on each cluster was more accurate (Fig. 3D, far right panel). If the two­-cluster 3D-NE model approach generates overlapping volumes, then the algorithm subtracts surface and volume of the intersection. During late anaphase, a significant number of cNPCs were also detected in a long nuclear envelope tube connecting the two nuclei (extended hourglass shape). Such a configuration was explored by defining a third cluster of cNPCs (Fig. 3E). Therefore, by considering one, two or three cNPCs clusters, nuclei at each stage of the cell cycle can be fitted using the 3D-NE model.

### Quantification of the nuclear geometry during the cell cycle

**Fig. 4.**
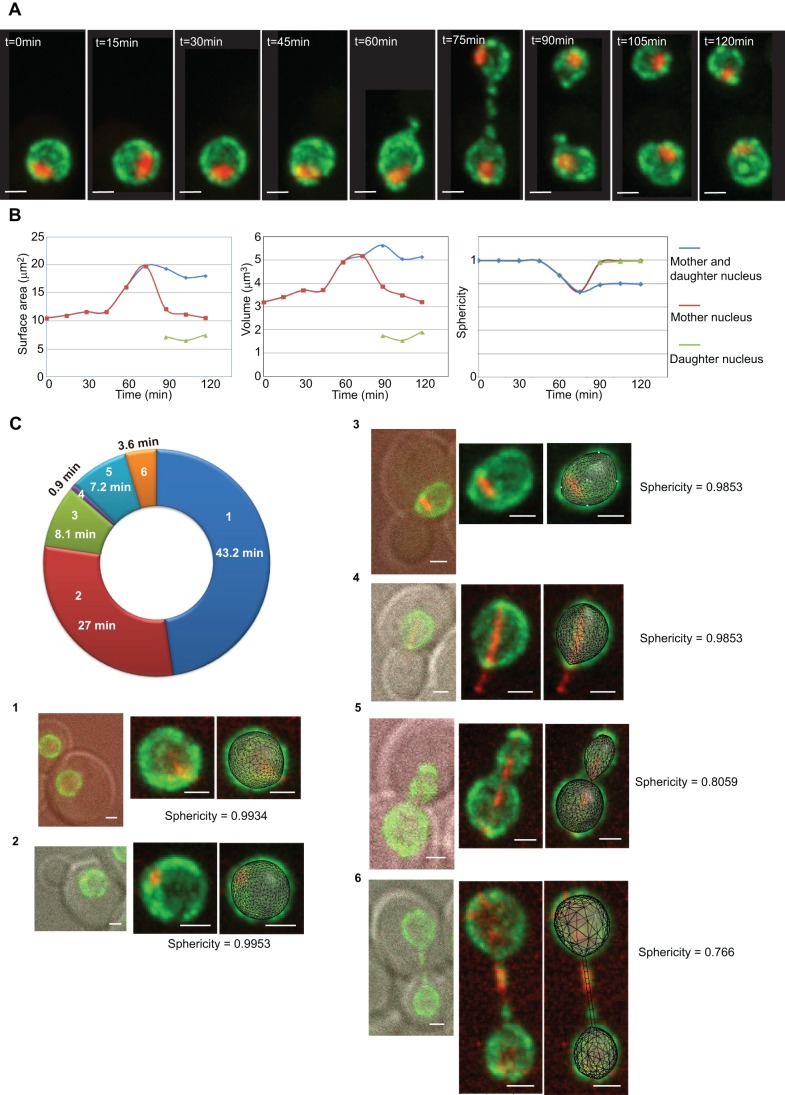
**Living yeast nuclear geometry during the cell cycle.** (A) Time course during a complete cell cycle of a single cell immobilized in a microfluidic device. NPCs in green and nucleolus in red (maximum intensity projections of a 3D image stack). Strain yCNOD99-1a. Note that a black rectangle of equivalent size was used as a background for cropped images. (B) The fitted nuclear envelope based on 3D-NE model for the nuclei in the different cell cycle phases. The surface of the nuclear envelope and the volume of nuclei allowed calculation of sphericity. (C) 3D-NE model fitting of different nuclear shapes (stages 1 to 6) throughout the cell cycle. The cell cycle is represented as a circle; the percentage of cells in each cell cycle phase from a large population was converted to duration (min). For each stage (panels 1 to 6), the DIC and the fluorescent (GFP–Nup49 and Bim1–tDimerRFP) pictures are displayed (stages 1 to 6). Strain yCNOD203-1a. The fitting using the 3D nuclear envelope model is also shown for each stage and was used to calculate sphericity. Scale bars: 1 µm.

Using the improved 3D-NE model described above, we investigated the nuclear size and shape modifications throughout the cell cycle. To avoid any potential perturbations of the nucleus caused by hormonal synchronization, living yeast cells were first observed in a microfluidic setup for an entire cell cycle (∼90 min), with image acquisitions every 15 min (Fig. 4A). Using our algorithm, we could accurately estimate surface and volume of nuclei throughout the cell cycle, allowing calculation of sphericity (Fig. 4B). Sphericity is defined as the ratio of the surface of a theoretical sphere having the same volume as the analyzed nucleus, to the measured surface of the considered nucleus (Wadell, 1935) (see Materials and Methods). Here, we analyzed 20 cells using the microfluidic setup. During interphase, nuclei increased in volume from 3 to 4.2 µm^3^. Nuclear division resulted in a new daughter cell nucleus of 2 µm^3^, and a mother cell nucleus of larger size (3.4 µm^3^) in agreement with previous reports (Fig. 4B; Table S1) (Heun et al., 2001). Sphericity is high in interphase (0 to 45 min), and is largely reduced in mitosis (60 to 90 min). After mitosis, mother and daughter nuclei are both close to a sphere (Fig. 4B, right panel). Acquisition time interval of 15 min in the microfluidic setup was established to reduce bleaching of fluorophores but increased the probability of missing some transient nuclear shapes (Yeh et al., 1995). To overcome this limitation, we analyzed nuclei in large asynchronous cell populations. Indeed, the fraction of cells in one cell cycle phase in a large population is proportional to its duration; we thus converted percentage of cells in each phase directly to time in minutes. We could classify six stages along the 90 min cell cycle and measured sphericity for each stage (Fig. 4C). Cells in G1 (stage 1) and in S phase (stage 2) have quasi-spherical nuclei (sphericity>0.99). In late S, rapid (8 min) extension of the intranuclear microtubule spindle provokes a small compression of nuclei (stage 3). Prior to nuclei entering through the bud constriction, stage 4 was characterized by elongated nuclei (1 min) along spindle axis. From stage 1 to 4, fitting the 3D-NE model with one cluster was appropriate. Once nuclei entered through bud constriction (stages 5 and 6), two or three clusters had to be used to fit the nuclear envelope. In stage 5, sphericity is reduced by bud construction and spindle elongation (from 0.98 to 0.8). At the end of mitosis, hour-glass shaped nuclei were elongated with a tube connecting the two nuclei (sphericity <0.8).

In conclusion, our approach allowed the quantification of cell cycle nuclear variations in *S. cerevisiae* assessed in single living cells and in large cell populations.

### Geometry of interphasic nuclei in different metabolic conditions

**Fig. 5.**
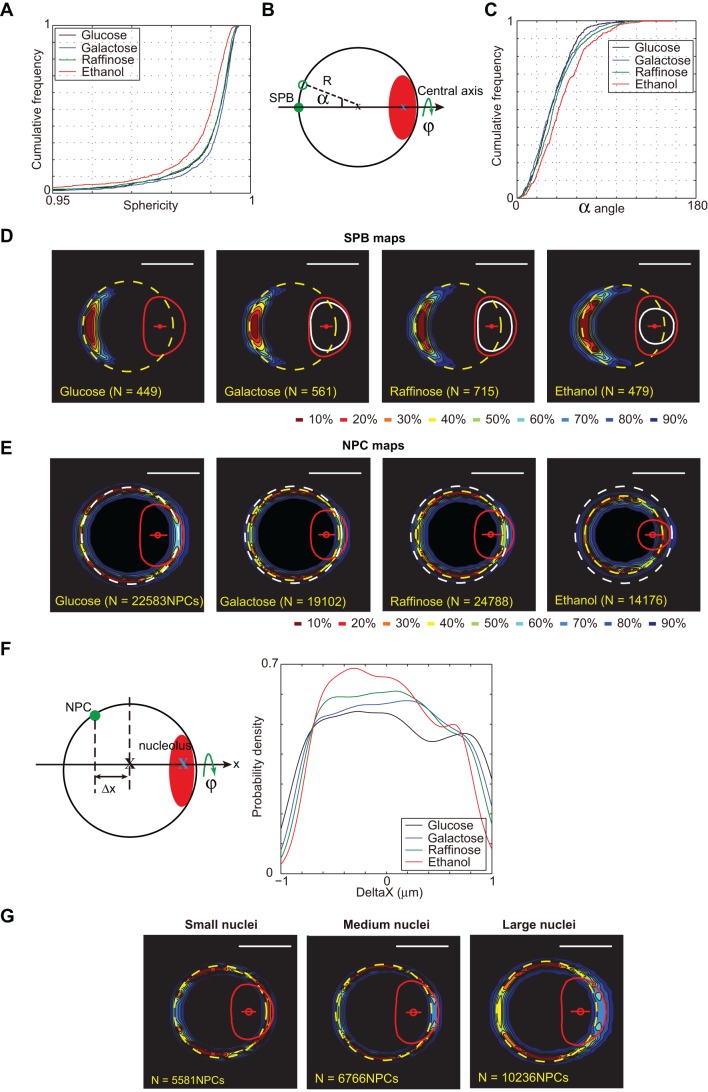
**The nuclear geometry according to the carbon sources.** (A) Cumulative distribution of sphericity of the interphase nuclei cultured in different carbon sources. Strain yCNOD99-1a. (B) Cylindrical coordinates system with an oriented axis in which the position of the SPB is described by its distance from the nuclear center (R) and the angle from the central axis (α). Nucleolus is displayed in red. Angle φ represent rotation around central axis. (C) Cumulative frequency of the angle α between SPB and the central axis. Strain yRW11-1a. (D) SPB probability density maps based on analysis of nuclei comparing glucose with different carbon sources containing media. In glucose: dashed yellow circle, nuclear envelope determined according to the 3D-NE method; red curve, median nucleolus; red dot, median nucleolar centroid. Compare nucleolar size in glucose (red) with nucleolar size in other carbons sources (white). *N* represents the number of nuclei used to generate the cumulative percentage maps. (E) NPCs probability density maps based on analysis of nuclei in exponential phase cells growing in different carbon sources. Strain yCNOD99-1a. Compare median nuclear size in glucose (white dashed circle) with other carbon sources (yellow dashed circles). *N* represents the number of cNPCs used to generate the cumulative percentage maps. (F) Plotted variation of NPC density along the central axis in response to different carbon sources. (G) Heterogeneity of NPC distribution in interphasic cells. Nuclei were sorted according to their size (1/3 small, 1/3 medium, 1/3 large nuclei. Strain yCNOD99-1a. Scale bars: 1 μm.

It has been reported that nuclear size is reduced when the carbon source is changed from glucose to less favorable ones such as galactose, raffinose or ethanol (Jorgensen et al., 2007). We tested if we could measure such reduced nuclear size and any potential perturbations of the nuclear envelope shape for these different diets. Each carbon source impacted the cell doubling time: 90 min in 2% glucose, 120 min in 2% galactose, 130 min in 2% raffinose and 220 min in 3% ethanol. Sphericity stayed high in all conditions, but was reduced as doubling time increased (Fig. 5A). When changing from most to less favorable carbon sources, we could measure a gradual ∼twofold reduction of the nuclear volume, and an associated fivefold reduction of the nucleolar volume (Table S2).

Such drastic modification of the nucleoplasm/nucleolus ratio might affect internal nuclear organization. In glucose, the SPB and the nucleolus are roughly opposed in interphasic cells, defining a central axis (alignment of the SPB, nuclear center and nucleolar centroid). We then explored the 3D geometry of the SPB relative to the nucleolus when the nucleolar volume was modified. We described the SPB and the nucleolus positions using probability density maps (Berger et al., 2008; Therizols et al., 2010). Briefly, we made use of nucleoli labeled with the mCherry-tagged Nop1 protein (yeast ortholog of fibrillarin). An abundant SPB constituent Spc42 tagged with GFP was used to detect the SPB, which could be distinguished from NPC signal in the same green channel owing to to its high fluorescence. The SPB is duplicated during S phase and the two SPBs are pulled apart during G2/M. Only cells in G1 and early S phase with a single spot corresponding to the SPB were considered for analysis. The SPB was positioned relative to two reference points: the nuclear center and the centroid of the nucleolus. This approach enabled us to define a cylindrical coordinates system with an oriented axis in which we described the position of the SPB by its distance from the nuclear center (R) and the angle from the central axis (α; Fig. 5B). In galactose or raffinose, the SPB was indeed opposite to the nucleolus as it was in glucose, with a median angle α of 30°. When the less favorable carbon source ethanol was used, this angle significantly increased to 40° (Fig. 5C). We next generated a probability density map of SPB distribution using the nuclear and nucleolar center as landmarks. To align the all nuclei analyzed in the same coordinates system, we translated all nuclear centers to the origin and rotated them around the origin so that nucleolar centroids (secondary landmark) became aligned on the *x*-axis. This allowed rotation of SPB positions around the central axis (Fig. 5B, ϕ=angle around central axis) and into a single plane (ϕ=0, cylindrical projection), essentially without loss of information. Kernel density estimation was then used to define a SPB density map, similar to the previously described probability density map of genomic loci (Berger et al., 2008; Therizols et al., 2010). On the SPB density map (Fig. 5D), the yellow dashed circle represents the median nucleus periphery and the median nucleolus is displayed as solid red (glucose) or orange (other carbon sources) curves. The SPB remains opposed to the nucleolus, but nucleolus was reduced massively, up to fivefold (compare red with orange solid lines) (Fig. 5D).

We then decided to evaluate the distribution of detected cNPCs using the same central axis, to generate an NPC map using similar kernel density distribution. Plotting NPC maps, we could quantify the probability density of cNPCs in the nuclear envelope of yeast growing in glucose, galactose, raffinose or ethanol (Fig. 5E). On this NPC density map, the white dashed circle represents the median nucleus periphery in glucose medium and the yellow dashed circles correspond to median nucleus in other carbon sources. As previously described, nucleus size was drastically reduced when the growth rate was reduced (compare yellow with white dashed circle; see also Table S2) (Jorgensen et al., 2007). cNPC density along the nuclear envelope was non-uniform. To quantify differences in cNPC density between the yeast growing in different carbon sources, we plotted variation of cNPC density along the central axis (Fig. 5F). In all carbon sources tested, detected cNPCs appeared slightly depleted in the nuclear envelope at the nucleolus–nucleoplasm border. In glucose and ethanol, cNPC density slightly increased in the nuclear envelope flanking the nucleolus. The cNPC non-uniform density along the nuclear envelope could reflect either a non-uniform distribution on each individual cell, or aggregation of heterogeneous nuclei in the population. In order to explore this possible heterogeneity, we then sorted nuclei from cells growing in glucose according to their volume in three classes (small, medium and large), and plotted the three corresponding NPC maps (Fig. 5G). In the nuclear envelope flanking the nucleolus, the cNPC density was higher in small nuclei than in large nuclei. At the SPB (opposed to the nucleolus) similar variation of cNPC density was also detected across the range of nucleus sizes. Similar heterogeneity was observed in populations growing in galactose and raffinose media (data not shown).

In conclusion, when changing carbon source, yeast nuclei in interphasic cells remain largely spherical. However, in less favorable carbon sources, sphericity is reduced and SPB position deviates from the nuclear–nucleolar central axis. Further, we could always observe a depletion of detected cNPCs at the interface of nucleoplasm and nucleolus. cNPC density in the nuclear envelope flanking the nucleolus varies with nuclear size. Therefore, reduction of the nucleolar volume correlates with reorganization of SPB position and modification of cNPC density along the nuclear envelope.

### Exploration of the nuclear envelope during quiescence

**Fig. 6.**
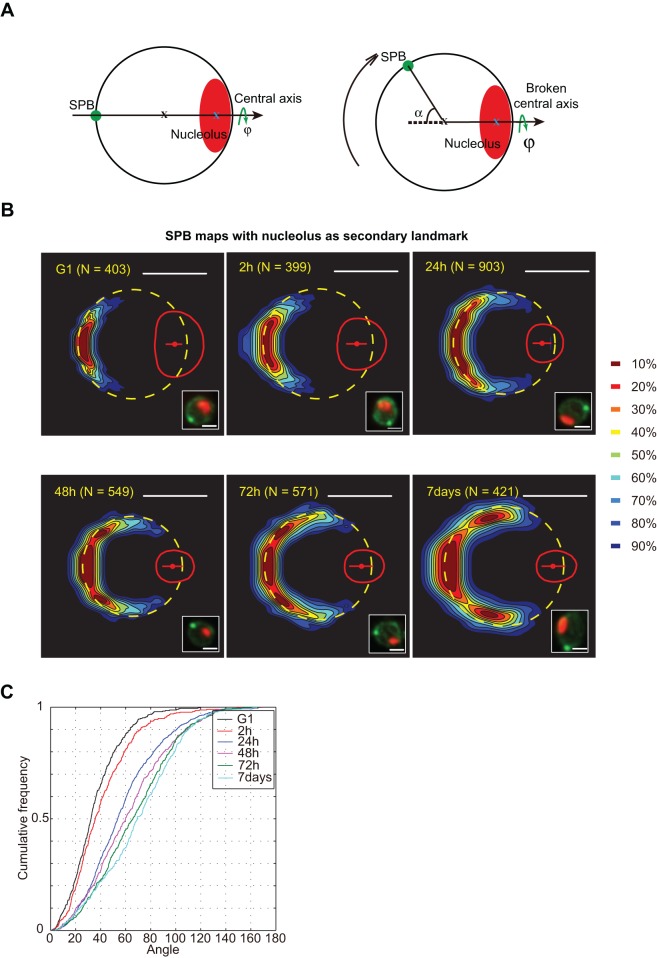
**The reorganization of the nuclear central axis during quiescence.** (A) The nuclear central axis (SPB–nuclear-center–nucleolar-centroid) is broken after the cells enter quiescence. Red ellipse, nucleolus; green circle, SPB; black cross, nucleus centroid; blue cross, nucleolus centroid; α, angle of SPB to the nuclear–nucleolar­centroid axis. (B) SPB probability density maps based on analysis of nuclei after indicated time of starvation (see Materials and Methods). Representative fluorescent pictures (GFP–Nup49, Spc42–GFP and mCherry–Nop1) are displayed. Scale bars: 1 μm. (C) Cumulative frequency of the angle α upon incubation in glucose-depleted medium (from 2 h to 7 days). Strain yRW11-1a.

We next explored nuclear size and shape during the establishment of quiescence, which has a drastic impact on the nucleolar size. Upon nutrient depletion, ribosome biogenesis is quickly repressed, resulting in the compaction of the nucleolus (Tsang et al., 2003). In quiescent cells, following an extended period of nutrient depletion, Sagot's laboratory have shown that the SPB assembles a long monopolar array of stable microtubules associated with a displacement of the nucleolus (Laporte et al., 2013). As shown in Fig. 6A, in our quantitative approach this observation would lead in an increase of the α angle. We determined the SPB density map upon glucose depletion and establishment of quiescence (from 2 h depletion to 7 days; Fig. 6B). During establishment of quiescence, the nucleolar volume decreased and the SPB distribution deviated progressively from the central axis. The α angle progressively increased during quiescence establishment, with median distribution increasing from 30° in exponentially growing cells to 70° after 7 days of nutrient depletion (Fig. 6C).

**Fig. 7.**
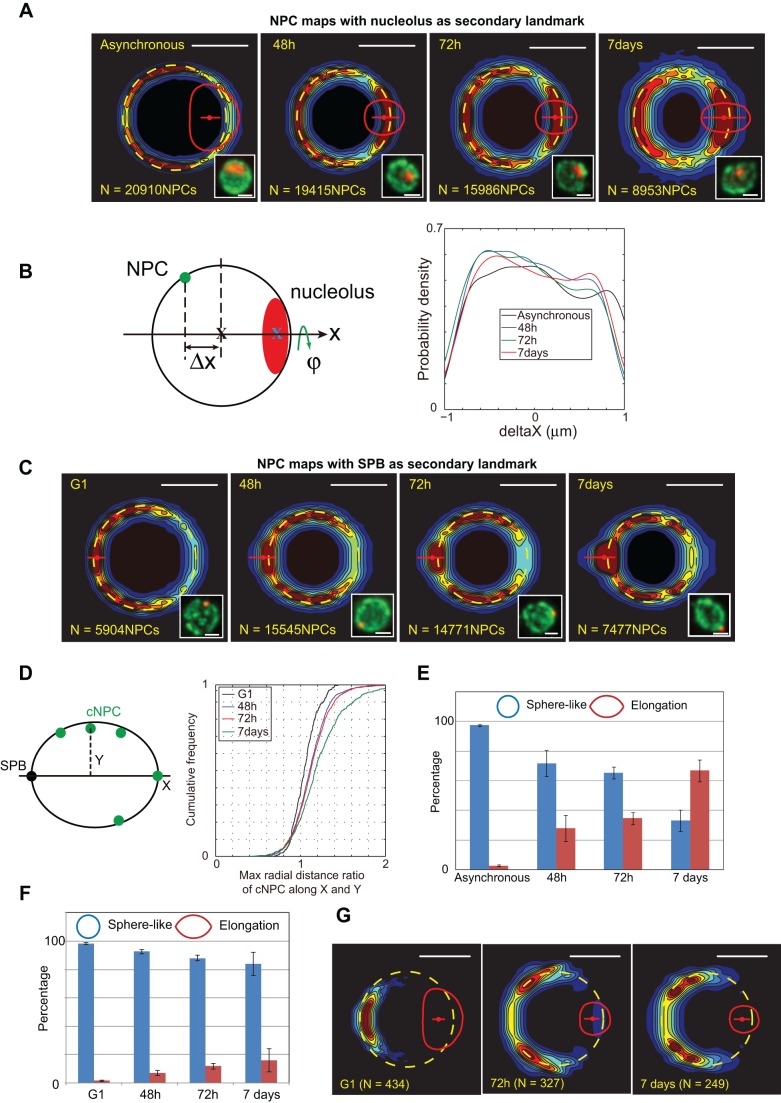
**The nuclear envelope structure and NPC distribution during quiescence.** (A) NPC probability density maps using the nucleolus as a secondary landmark upon time-progressive incubation in glucose-depleted medium. Representative fluorescent pictures (GFP–Nup49 and mCherry–Nop1) are displayed. Strain yCNOD99-1a. (B) Plotted variation of NPC density along the central axis during progressive starvation. (C) NPC probability density maps using SPB as a secondary landmark upon time­-progressive incubation in glucose-­depleted medium. Small red dot, SPB median position. Representative fluorescent pictures (GFP–Nup49 and SPC42–mRFP) are displayed. Strain yRW9-1a. (D) Maximum radial distance ratio of cNPC along *x* and *y* axis. Strain yRW9-1a. (E) After the cells enter quiescence, the percentage of different nuclear geometries at incubation times in carbon-depleted medium. Strain yCNOD99-1a. (F) Percentage of elongated nuclei versus sphere-like nuclei in the *dyn1*Δ mutant after 48 h to 7 days of carbon depletion. Strain yRW19-1a. Results in E,F are represented as mean±s.e.m. (G) SPB probability density maps based on analysis of nuclei from *dyn1*Δ mutant cells after indicating time of starvation. Strain yRW20-1a. Scale bars: 1 μm.

We next evaluated the distribution of cNPCs along the nuclear envelope during establishment of quiescence. Importantly, because the SPB and the nucleolus are not aligned during this process, we performed NPC mapping using either the nucleolus (Fig. 7A) or SPB (Fig. 7C) as a secondary landmark. Before depletion, the NPC map accurately defined the median nucleus. After 7 days, cNPC distribution was spread around the median nucleus, reflecting the great heterogeneity of the nuclear envelope size and/or shape amongst quiescent cells (Fig. 7A,C). However, some stereotypical patterns in cNPC distribution were clearly visible; depletion of detected cNPC at the nucleolus–nucleoplasm interface, and the cNPC concentration toward the nucleolus observed in optimal growth conditions were strengthened during starvation. This distribution was illustrated by plotted variations of cNPC density along the central axis (Fig. 7B). Using the SPB as a secondary landmark (Fig. 7C), NPC maps revealed an increased number of cNPCs detected in close proximity to the SPB during establishment of quiescence. Indeed, the maximum radial distance ratio of cNPC along the *x* and *y* axes gradually increased during establishment of quiescence (Fig. 7D). We also visually detected a change of nuclear shape from spherical to elongated in more than 60% of cell nuclei (Fig. 7E). We hypothesized that the stable monopolar array of microtubules could displace the nucleolus and modify the nuclear envelope shape (Laporte et al., 2013). To test which of these modifications was caused by starvation, or by the long monopolar array of stable microtubules, we evaluated nuclear shape and nucleolar position in a *dyn1*Δ mutant, in which the microtubule spindle is disrupted (Laporte et al., 2013). As expected, an elongated nuclear shape was not observed in the mutant cells (Fig. 7F). Surprisingly, SPB deviation from the nuclear–nucleolar-centroid axis that increased during establishment of quiescence was independent of spindle establishment (Fig. 7G). In conclusion, we detected a loss of axial symmetry (the alignment between the SPB, nucleus and nucleolar centers) in quiescent cells. Furthermore, stable microtubules were involved in nuclear envelope deformation, but not in the loss of axial symmetry.

### Modification of the nuclear envelope shape during G1 cell cycle arrest

**Fig. 8.**
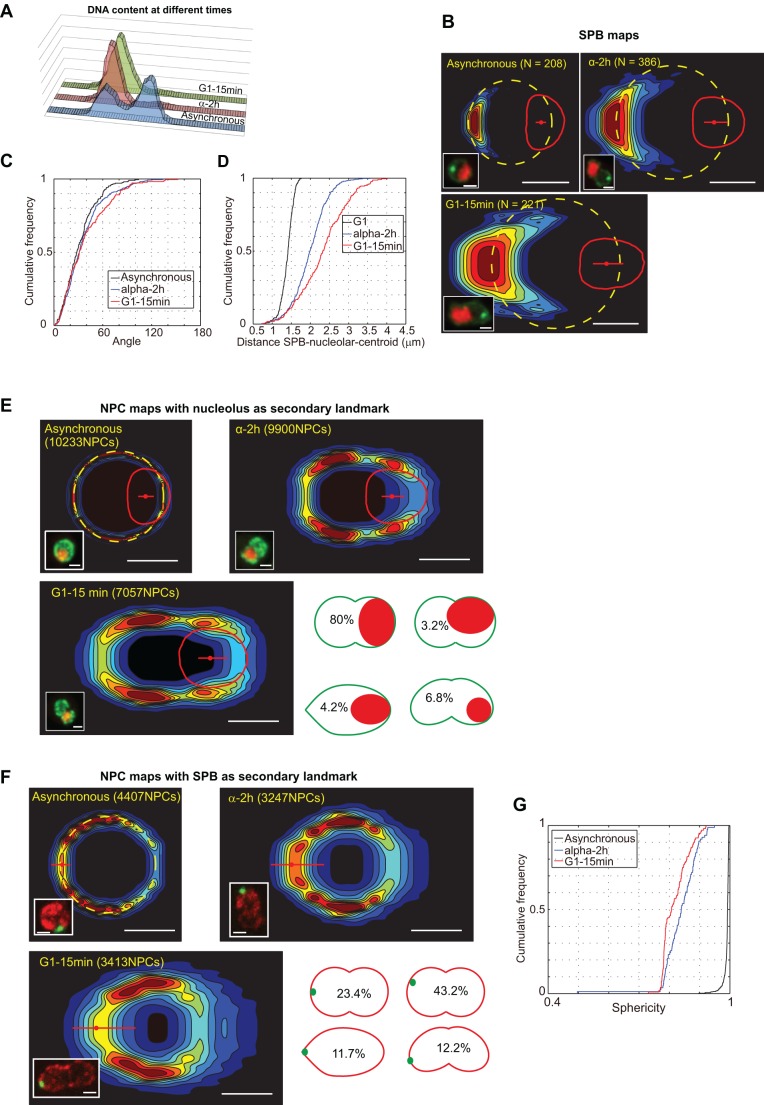
**Modification of the nuclear envelope after treatment with alpha factor.** (A) DNA content in asynchronous culture after 2 h of alpha factor treatment and after alpha factor removal determined by flow cytometry. Strain yCNOD99-1a. (B) SPB probability density maps before and after treatment with alpha factor using the nucleolus as a secondary landmark. Representative fluorescent pictures (GFP–Nup49, SPC42–GFP and mCherry–Nop1) are displayed. Strain yRW11-1a. (C) Cumulative frequency of the SPB–central-axis angle. (D) Cumulative frequency of the distances from the SPB to the nucleolar centroid. (E) NPC probability density maps in an asynchronous population (left map), after 2 h of alpha factor blocking (right map) and 15 min after release in G1 (bottom map), using the nucleolus as a secondary landmark. Representative fluorescent pictures (GFP–Nup49 and mCherry–Nop1) are displayed. Strain yCNOD99-1a. Drawings depict the different nuclear shapes and the position and size of the nucleolus after 2 h treatment with alpha factor. (F) NPC probability density maps before and after treatment with alpha factor using the SPB as a secondary landmark. Representative fluorescent pictures (SPC42–GFP and Nup57–tDimerRFP) are displayed. Strain yRW10-1a. Drawings depict the percentage of different nuclear geometries after 2 h treatment with alpha factor. (G) Cumulative distribution of sphericity after 2 h of alpha factor blocking and 15 min after release in G1. Scale bars: 1 μm.

In all tested conditions (variation of carbon sources, and quiescence), the nucleolar volume was reduced. We thus analyzed nuclear envelope geometry in G1 arrested cells in which the nucleolar volume is increased (Stone et al., 2000). A well-established method to analyze cell cycle progression is to synchronize cells in bulk culture. Asynchronous cells (*MATα*) were blocked in G1 using alpha factor treatment, causing the fraction of cells in G1 to progressively increase. Arrest lasting for more than an entire cell cycle progression resulted in a cell population with almost all cells in G1 phase. A quick wash to remove the alpha factor allowed cells to progress synchronously through the cell cycle as illustrated in Fig. 8A. However, cell cycle arrest is known to influence nuclear envelope morphology; upon alpha factor treatment, budding yeast nuclei adopt an unusual dumbbell shape, reflecting a spatial separation of chromosomal and nucleolar domains and an increase of the nucleolar volume (Stone et al., 2000). SPB density map analysis revealed that SPB maximum density remained opposed to the nucleolus during alpha factor treatment, but had a considerably broader distribution (Fig. 8B). Although the angle of the SPB to the central axis was not affected during the treatment (Fig. 8C), the distance from the SPB to the nucleolar centroid was significantly increased and variable from cell to cell (Fig. 8D). An NPC map using the nucleolar centroid as a secondary landmark allowed us to visualize the stereotypical dumbbell shape previously reported, and the twofold increase in nucleolar volume (Stone et al., 2000) (Fig. 8E). In 80% of cells, the nucleolus was at the center of one lobe of the dumbbell. As in asynchronous cells, we detected cNPC depletion at the nuclear envelope interface between nucleoplasm and nucleolus. Using the SPB as a secondary landmark, NPC mapping revealed a fuzzy distribution toward the nucleolus (Fig. 8F). Visual inspection of the SPB position along the nuclear envelope showed a deviation from the central axis of the dumbbell-shaped nuclei in more than 55% of the cells (sum of the two classes with SPB away from the central axis; Fig. 8F). It has been reported that microtubules emanating from the SPB are not involved in such nuclear reorganization (Stone et al., 2000). We propose that heterogeneity in measured distances between the SPB and the nucleolus, and in the position of the SPB in dumbbell-shaped nuclei resulted in a blurred NPC density map (Fig. 8F). We quantitated this heterogeneity when measuring sphericity, which decreased to a median value of 0.85 upon treatment, and an increased standard deviation (Fig. 8G).

In conclusion, upon alpha factor treatment, increased nucleolar volume did not modify the nuclear central axis, but did specifically change nuclear envelope morphology flanking the nucleolus and correlated with an increased nuclear envelope surface.

## DISCUSSION

In this paper, we explore the geometry of *S. cerevisiae* nuclei in living cells using high-resolution microscopy detection of closely spaced nuclear pore complexes (cNPCs) embedded in the nuclear envelope. Firstly, nuclear geometry was extrapolated from analysis of single living cells throughout the cell cycle and in cell populations. Heterogeneity of the 3D nuclear geometry could be quantified using the developed ‘NucQuant’ algorithm. Secondly, the aggregation of numerous aligned individual cNPCs detected in nuclei of large living cell populations allowed us to generate NPC density maps. Using either the SPB or nucleolus as landmarks, the maps revealed an asymmetric distribution of the cNPCs. We also generated SPB density maps to explore, with robust statistics, the distribution of SPB positions relative to the nucleolus. Moreover, analysis of starving cells with or without stable microtubule bundles and G1-arrested cells revealed modifications of the nuclear shape with stereotypical deformations of the nuclear envelope toward the nucleolus.

### The need for new approaches to evaluate nuclear geometry in living cells

Nuclear envelope morphology is dynamic and, owing to the limited resolution of fluorescent microscopy, is mostly described qualitatively. Most of the existing measurements are diffraction-limited, i.e. 200 nm lateral (*x*–*y*) and 500 nm axial (*z*) resolution, and often biased by optical spherical aberrations. Other techniques with higher resolution have been reported. Initially, transmission electron microscopy (TEM) performed on ultra-thin sections of yeast nuclei revealed a heterogeneous appearance of nuclear envelopes, probably mostly resulting from chemical fixation artefacts (I.L.-S., unpublished results). When using sample-preserving fixation, such as high-pressure cryo-fixation followed by cryo-substitution, TEM of ultra-thin (60–80 nm) sections of nuclear envelope showed a smooth double membrane envelope with circular or ellipsoidal contours. When performed on ultra-thin serial sections of an entire nucleus, TEM became directly informative of the nuclear shape and volume. However, data collection of serial sections analyzed by TEM is a time-consuming technical performance and has been reported for sampling of less than 100 nuclei (Winey et al., 1997). Innovative imaging techniques, relying on cryo-fixation but with less tedious exploration of 3D space, as high-voltage scanning transmission electron tomography of thick sections (Murata et al., 2014), focused ion beam combined with scanning electron microscopy (FIB–SEM) (Wei et al., 2012), and soft-X-ray tomography (Larabell and Le Gros, 2004) are very promising. However, they are not compatible with *in vivo* imaging, and they have not yet been combined with high-throughput image analysis algorithms required to extract statistically robust datasets. Recently, a 3D membrane reconstruction approach that used fluorescently tagged nuclear envelope or endoplasmic reticulum membrane marker proteins was published to precisely map the nuclear volume (Zhao et al., 2016). Here, we show that super-resolution localization microscopy of cNPC allow both accurate nuclear size and shape determination and proper correction of spherical aberration along the *z* axis. These two methods are each bringing different information on nuclear envelope shape and NPC distribution.

### Discontinuous increase of nuclear size along the cell cycle

*Saccharomyces* *cerevisiae* nuclei in interphasic cells appear quasi-round when examined by fluorescence microscopy. Quantifying their sphericity in 3D with the NucQuant approach, we confirmed this observation. Moreover, exploring heterogeneity in interphasic cell nucleus, we could also recapitulate previously observed modifications of the nuclear shape in interphase, triggered by microtubule spindles prior to mitosis (Yeh et al., 1995). However, proportions of these clamped and elongated nuclei in asynchronous populations were low, reflecting very transient states. Additionally, following a single cell nucleus over time using a microfluidic setup allowed us to measure the nuclear envelope surface and the nuclear volume, showing a slight deviation from a sphere in G1 and S phases. Previous studies have suggested a continuous increase of the nuclear volume during the cell cycle (Jorgensen et al., 2007). By contrast, we observed a discontinuous increase of the nuclear envelope surface; at mitotic onset (60 min), we detected a fast and significant nuclear envelope expansion, whereas the nuclear volume slightly increased from G1 to M. The measured timing of this rapid nuclear envelope surface increase is fully compatible with the activation timing of polo kinase Cdc5 described to be required for nuclear envelope expansion at mitotic onset (Walters et al., 2014).

### Non-homogeneous distribution of closely spaced NPC (cNPC) near the SPB and nucleolus

We observed non-uniform distribution of detected cNPCs along the nuclear envelope. The analysis of size-sorted interphasic nuclei showed that cNPC density in the nuclear envelope near the SPB or flanking the nucleolus was different in small round early G1 nuclei versus large round late S nuclei. In quiescence, depletion of cNPCs at the nucleolus–nucleoplasm interface, and the cNPC concentration toward the SPB and toward the nucleolus observed in optimal growth conditions, were strengthened. This might reflect different physical properties of the nuclear envelope near the SPB and at the nucleolus. A relationship between the SPB and NPCs has been previously described: when the SPB is ready to be duplicated in late G1 (i.e. small nuclei or quiescent cells), NPCs are frequently detected near the SPB (Winey and O'Toole, 2001). The authors proposed that the local nuclear envelope bending by NPCs was required for insertion of the newly synthetized SPB. The variation that we measured in cNPC concentration near the SPB could reflect this involvement of NPCs in SPB duplication. However, such specific enrichment is not detectable in artificially arrested cells in G1.

Specific interplay between the nuclear envelope, the nucleolus and NPCs is also known. Nuclear envelope flanking the nucleolus is depleted of NPC-associated proteins such as Mlp1,Mlp2 (Galy et al., 2004), Pml39 (Palancade et al., 2005), and of the inner nuclear membrane protein Esc1 (Taddei et al., 2004), suggesting that the NPCs and the inner membrane flanking the nucleolus are specialized. The nuclear envelope flanking the nucleolus is also known to correlate with a local modification of the nuclear envelope rigidity (Witkin et al., 2012). Is the nucleolus physically connected to the nuclear envelope? Previous work has established a connection between the nuclear envelope and the nucleolus via INM non-NPC-associated proteins, Nur1 and Heh1, distributed through the entire nuclear envelope and involved in peripheral tethering of ribosomal DNA (Mekhail et al., 2008). We propose that NPCs are physically anchored to the nucleolus. Indeed, we observed that cNPC concentration at the nuclear envelope flanking the nucleolus or at the nucleolus–nucleoplasm interface was highly variable according to cell physiology. Nucleolar volume rapidly changes following inhibition or activation of ribosome biogenesis (Trumtel et al., 2000). Upon quiescence caused by starvation or a change of carbon source, nucleolar volume decreased but the nucleolus remained associated with the nuclear envelope. Therefore, a significant fraction of the nuclear envelope at the nucleolus–nucleoplasm interface lost its nucleolar connection. A physical connection of NPCs with the nucleolar component would explain their local concentration at the remaining nuclear envelope flanking the nucleolus and their depletion at the nuclear envelope flanking the nucleolus–nucleoplasm interface. Further, such observations suggest that physical association between NPCs flanking the nucleolus and nucleolar components is dependent upon ribosome biogenesis inhibition.

### Nuclear geometry alteration

One important parameter in modeling genome organization in *S. cerevisiae* is the confinement of the genomic material in the nucleoplasm (Tjong et al., 2012; Wong et al., 2012). The SPB is roughly opposite to the nucleolus in interphase, and the nucleolus forms a nuclear compartment that excludes 90% of the genomic material. Thus, a central axis was used to define yeast nuclear geometry (angle α=0; SPB–nuclear-center to nuclear-center–nucleolar-centroid) (Berger et al., 2008). Here, we observed that this central axis geometry was strongly correlated with nucleolar activity. When nucleolar activity was maximized (short cell cycle in rich glucose medium), deviation from ideal central axis organization was low (<30°). Upon G1 arrest, which stops cell growth but keeps a large nucleolar volume, this angle was unchanged. When nucleolar volume was reduced in conditions where growth rate was impaired using an unfavorable carbon source, the angle increased. Finally, in quiescent cells in which the nucleolar volume was further reduced, this angle increased to 70°. Moreover, this large modification of nuclear geometry in quiescence did not depend on stable long monopolar array of microtubules. Therefore, we propose that nucleolar size, and not growth rate directly, is involved in keeping internal geometry of the nucleus around the central axis.

### Conclusion

In conclusion, we have developed NucQuant, a set of computational tools to quantify the nuclear geometry (size and shape) of *S. cerevisiae*. The statistical robustness and accurate measurements obtained with this approach recapitulated known stereotypical rearrangements of the yeast nucleus, and uncovered heterogeneity of cNPC concentration along the nuclear envelope. A model of yeast chromosomes had already been computed based on geometry of round-shaped nuclei (mostly in G1 cells). Models of chromosomes in nuclei of quiescent cells did not take into account the modifications of the nuclear morphology as quantified in our work (Guidi et al., 2015). Quantification of stereotypical modifications of the nuclear morphology, observed when changing carbon source, upon quiescence or in G1-arrested cells, will now allow refinement of chromosome structure models by integrating changes in nuclear confinement defined by nuclear shape and size, and by modifications of the nucleolar compartment. Numerous observations point to a relationship between the nuclear size and shape and pathological processes or aging (Webster et al., 2009). However, apparent heterogeneity from nucleus to nucleus limits our ability to study mechanistic insight. Probabilistic density maps, presented here for a model organism, might drive future efforts for metazoan cell nucleus analysis, as already proposed for endomembrane organization (Schauer et al., 2010).

## MATERIALS AND METHODS

### Plasmids and yeast strains

Genotypes of the strains used in this study are described in Table S3. Plasmids used in this study are listed in Table S4. Yeast strains were constructed using homologous recombination as previously described (Longtine et al., 1998; Sung et al., 2008).

### Fluorescence and electron microscopy of yeast cells

Yeast media were used as previously described (Rose et al., 1990). A home-made PDMS chamber connected to a microfluidic pump (Fluigent S.A.) allowed trapping of cells under a constant flow of growth medium for more than 2 h. Confocal microscopy was performed as previously described (Albert et al., 2013). Electron microscopy was performed as previously described (Albert et al., 2011).

### NucQuant: post-acquisition correction of *z* aberration

Confocal images were processed and analyzed with MATLAB (MathWorks) script NucQuant, a modified version of nucloc (http://www.nucloc.org/).

We calculated the distances between the nuclear center and the cNPCs. For each nucleus these distances were normalized by nucleus mean radius (R). This distribution was best fitted by the second-degree polynomial curve: ax^2^+bx+c. This equation could be used to correct the aberration along the *z* axis: d_correct_/R=d/R−(ax^2^+bx).

### NPC clustering by k-means

To reconstruct the nuclear envelope, we measured the distribution of NPCs in the clusters. After extracting the NPC positions and correcting the *z* aberration (*x_i_, y_i_, z_i_*), we used the *k*-means clustering method with Euclidian squared distance (MATLAB) to group the NPCs in different clusters (*C^k^*).

### Spline-NE

Clustering allowed us to distribute detected NPCs in each cluster *(x^k^_i_, y^k^_i_, z^k^_i_)_i=1..N_*, where *N* is the size of cluster *C^k^*. The first configuration of the nuclear envelope represented by the cluster *C^k^* is given by the polyhedral patch of the set *(x^k^_i_, y^k^_i_, z^k^_i_)_i=1..N_*. As the size of *C^k^* is very low for a smooth surface, we performed refining three times using an existing method (Shirman, 1990). This spline interpolation give us the refining sets *(x^k^_i_, y^k^_i_, z^k^_i_)_i=1..N3_* and *(x^k^_i_, y^k^_i_, z^k^_i_)_i=1..N4_* to make the surface more smooth.

### 3D-NE

The first configuration of the nuclear envelope represented by the cluster *C^k^* is given by the patch of the set *(x^k^_i_, y^k^_i_, z^k^_i_)_i=1..N_*. In this patch, the surface of the nuclear envelope consists of many Delaunay triangulations which were formed by connecting the neighboring three NPCs. For one-time refining, we generated one anchoring point of the nuclear envelope for each Delaunay triangulation. The new point in the direction from the cluster center to each Delaunay triangulation mass center, and the distance from the new point to the cluster center is the mean distance of these three NPCs to the cluster center (Fig. 3C). After refining three times, we obtained enough points to generate a precise envelope.
